# *Kaempferia parviflora* Extract Inhibits STAT3 Activation and Interleukin-6 Production in HeLa Cervical Cancer Cells

**DOI:** 10.3390/ijms20174226

**Published:** 2019-08-29

**Authors:** Benjamart Suradej, Siriwoot Sookkhee, Jukreera Panyakaew, Pitchaya Mungkornasawakul, Nitwara Wikan, Duncan R. Smith, Saranyapin Potikanond, Wutigri Nimlamool

**Affiliations:** 1 Department of Pharmacology, Faculty of Medicine, Chiang Mai University, Chiang Mai 50200, Thailand; 2 Graduate School, Chiang Mai University, Chiang Mai 50200, Thailand; 3 Department of Microbiology, Faculty of Medicine, Chiang Mai University, Chiang Mai 50200, Thailand; 4 Department of Chemistry, Faculty of Science, Chiang Mai University, Chiang Mai 50200, Thailand; 5 Environmental Science Research Center, Faculty of Science, Chiang Mai University, Chiang Mai 50200, Thailand; 6 Institute of Molecular Biosciences, Mahidol University, Salaya, Nakorn Pathom 73170, Thailand; 7 Research Center of Pharmaceutical Nanotechnology, Chiang Mai University, Chiang Mai 50200, Thailand

**Keywords:** anti-cancer, cervical cancer, *Kaempferia parviflora*, interleukin 6, STAT3 activation, EGFR phosphorylation, NF-κB

## Abstract

*Kaempferia parviflora* (KP) has been reported to have anti-cancer activities. We previously reported its effects against cervical cancer cells and continued to elucidate the effects of KP on inhibiting the production and secretion of interleukin (IL)-6, as well as its relevant signaling pathways involved in cervical tumorigenesis. We discovered that KP suppressed epidermal growth factor (EGF)-induced IL-6 secretion in HeLa cells, and it was associated with a reduced level of Glycoprotein 130 (GP130), phosphorylated signal transducers and activators of transcription 3 (STAT3), and Mcl-1. Our data clearly showed that KP has no effect on nuclear factor kappa B (NF-κB) localization status. However, we found that KP inhibited EGF-stimulated phosphorylation of tyrosine 1045 and tyrosine 1068 of EGF receptor (EGFR) without affecting its expression level. The inhibition of EGFR activation was verified by the observation that KP significantly suppressed a major downstream MAP kinase, ERK1/2. Consistently, KP reduced the expression of Ki-67 protein, which is a cellular marker for proliferation. Moreover, KP potently inhibited phosphorylation of STAT3, Akt, and the expression of Mcl-1 in response to exogenous IL-6 stimulation. These data suggest that KP suppresses EGF-induced production of IL-6 and inhibits its autocrine IL-6/STAT3 signaling critical for maintaining cancer cell progression. We believe that KP may be a potential alternative anti-cancer agent for suppressing cervical tumorigenesis.

## 1. Introduction

Although there is accumulating information on the biology of cervical cancer leading to advances in anti-cancer drug development, human cervical cancer is still one of the leading causes of death in women worldwide [1]. Interestingly, previous studies have revealed a strong link between cervical cancer and inflammatory cytokine signaling [2]. It has been reported that the high-risk human papilloma virus (HPV) 16 infection induces constitutive activation of signal transducers and activators of transcription 3 (STAT3) signaling [3]. HPV positive cancer cell lines such as HeLa (HPV18 positive) retained markedly higher levels of STAT3 phosphorylation at both Y705 and S727 residues compared with the HPV negative cell lines (C33A, DoTc2) [4]. 

Human IL-6 activates tyrosine kinase activity [5] and then triggers signaling cascades through the Janus family kinases (JAK)/STAT, Ras/MAPK, and PI3K/Akt pathways [6]. Specifically, upon interleukin (IL)-6 receptor activation, STAT3 is phosphorylated [7] and enhances cancer cell growth, survival, and immune evasion [3,8]. IL-6 has been proven to induce epithelial–mesenchymal transition in human cervical carcinoma cells via STAT3 activation [9]. Interestingly, IL-6 also enhances cervical cancer cell survival by upregulating the anti-apoptotic protein Mcl-1, which is mediated through the activation of the PI3K/Akt pathway [10]. Recently, it has been clearly demonstrated that the autocrine and paracrine actions of IL-6 are essential for STAT3 activation in HPV18-positive cervical cancer cell lines (SW756 and HeLa) [4]. In particular, this study revealed that activation of an IL-6 signaling axis drives the autocrine and paracrine phosphorylation of STAT3 within HPV-positive cervical cancers cells, and that activation of this pathway is essential for cervical cancer cell proliferation and survival.

Besides cytokines, binding of growth factors to their specific receptors can lead to the activation of STAT3, which typically involves phosphorylation of the tyrosine (Y) 705 of STAT3 [11]. STAT3 phosphorylation is primarily mediated by receptor-associated kinases such as Janus family kinases (JAK) and receptor tyrosine kinases including the epidermal growth factor receptor (EGFR) [12,13]. Phosphorylated STAT3 stimulates cell proliferation, apoptosis, immune regulation, and differentiation [14]. Furthermore, the expression of EGFR is associated with HPV infection [15]. Clinically, it has been shown that levels of EGFR and human papilloma virus (HPV)-E6 and E7 proteins are increased in the cervical epithelial cells of HPV-positive women with cervical cancer [16]. It indicates that HPV-positive cancer cells are sensitive to extracellular stimulation by EGF. This statement is supported by the study in CaSki and HeLa cells, showing that exogenous EGF stimulation enhances cell proliferation by activating EGFR and cyclin D1, which is independent of COX-2 levels, suggesting that the inhibitors of EGFR and cyclin D1 may be effective against cervical cancer cell proliferation. Specifically, the E5 protein of HPV type 16 binds to a subunit of the protein pump ATPase, which consequently leads to reduced degradation of EGFR, an increase in EGFR recycling, and overexpression of EGFR [17,18,19]. Moreover, expression of high-risk HPV E6 is associated with the increased level of EGFR [20]. Additionally, a high level of expression of EGFR was found to be correlated with a high density of Ki-67 positively-stained nuclei in severe dysplasia and carcinoma in situ, indicating a high proliferative state of cervical cancer cells [21]. Interestingly, a previous study has revealed that there is cross-talk between EGFR and IL-6 that drives oncogenic signaling [22]. This study demonstrated that newly synthesized IL-6 drives association of the IL-6 receptor and glycoprotein 130 (gp130) with EGFR, leading to EGFR-dependent rephosphorylation of STAT3. This phenomenon, where both IL-6 secretion and EGFR levels are often elevated, creates a perfect environment for cancer development and progression. Importantly, effective cytotoxic treatment options for advanced cervical cancer are exceedingly limited. The most commonly used cytotoxic therapy, cisplatin-based combination chemotherapy, has produced response rates ranging from 20% to 30% and overall survival rates of less than 10 months [23,24,25,26]. Because of this minimal success with cytotoxic therapies for cervical cancer, interest has increased in targeted therapeutics for the treatment of cervical cancer. For instance, Cetuximab, a chimeric immunoglobulin G2 monoclonal antibody that binds specifically to EGFR and competitively inhibits the binding of EGF and other ligands [27], has been developed to be used in patients with different cancers with high expression of EGFR including cervical cancers. Particularly, preclinical models have shown an exquisite sensitivity of cervical cancer to Cetuximab-mediated inhibition of tumor growth, and in this case, the antibody drug by itself does not directly induce cancer cell death, but suppresses the growth signaling [28]. On the basis of these previous studies suggesting the roles of IL-6 in cervical cancer development and progression, and the fact that cervical cancer cells have high EGFR expression, the discovery of agents that can be used in combination to suppress both IL-6/STAT3 and EGFR signal transduction pathways would possibly result in better treatment outcomes for cervical cancer. 

*Kaempferia parviflora* (KP) has been used as a folk remedy to treat various diseases including cancer. We previously demonstrated that the ethanolic extract of KP, with methoxyflavones as major constituents, exhibited strong anti-cancer activities against HeLa cervical cancer cells by suppressing the MAPK and PI3K/Akt signaling pathways stimulated with EGF [29]. Our previous study screened for the effects of KP at both toxic and non-toxic concentration ranges, and we successfully defined that KP at toxic concentrations induces HeLa cell death via intrinsic apoptotic pathway, and KP at non-toxic concentrations still possesses anti-cancer activities in which the extract does not directly induce cell death, but is able to suppress crucial molecular signaling in HeLa cervical cancer cells. One of our interesting findings was that KP at non-toxic concentrations interferes with EGF-stimulated growth and survival signal transduction pathways and inhibits cancer cell migration and invasion. However, the effects of KP at non-cytotoxic concentration on other important signaling pathways stimulated with EGF remain largely unexplored. In the current study, we continued our investigations to understand more about the anti-cancer activities of KP at various non-toxic concentrations by investigating the effects of KP on EGF-induced IL-6 production, and its relevant signaling pathways in an HPV18-positive cervical cancer cell line, HeLa. Because the extract at toxic concentrations can kill a majority of cells, and this eventually affects the level of intracellular proteins and the phosphorylation status, we thus studied the effects of KP at non-toxic concentrations to ensure that the reduction of all protein level and the phosphorylation status is not caused by cell death, but from the authentic properties of KP on interfering certain signal transduction pathways influenced by EGF. Thus, to further increase our understanding of its anti-cancer activities and to further support the use of KP in traditional medicine, we sought to expand our previous study by attempting to address whether KP has the ability to interfere with IL-6 production and secretion, as well as STAT3 activation in HeLa cells. We also defined a possible molecular mechanism of action of KP in suppressing IL-6/STAT3 signaling. Our study provides accumulated evidence that KP suppresses EGF-dependent growth/survival and IL-6/STAT3 signal transduction pathways, at least in part, through blocking the activation of EGFR. Also, results indicate that KP can impede the anti-apoptotic role of interleukin-6, which is normally required for maintaining cervical cancer cell survival [4].

As KP exhibits the ability to impede the tumorigenic influence of EGFR and IL-6 signaling in HeLa cells, we believe that KP could be a good candidate to be developed as an agent for treating HPV18-positive cervical cancer.

## 2. Results

### 2.1. Chemical Profile of Methoxyflavones in KP Extract and Effects of KP on IL-6 Production

**Figure 1:**
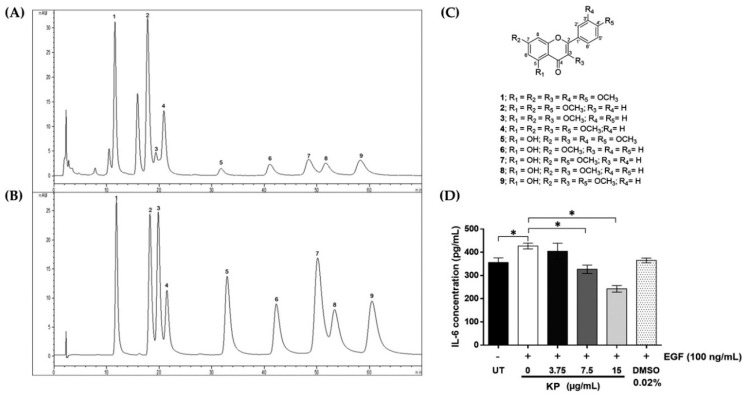
(**A**) High performance liquid chromatograph (HPLC) chromatogram of *Kaempferia parviflora* (KP) ethanolic extract; (**B**) HPLC chromatogram of mixed standard methoxyflavones 1 to 9; (**C**) the structure of standard compounds from KP; 3,5,7,3′,4′-pentamethoxyflavone (1), 5,7,4′-trimethoxyflavone (2), 3,5,7-trimethoxyflavone (3), 3,5,7,4′-tetramethoxyflavone (4), 5-hydroxy-3,7,3′,4′-tetramethoxyflavone (5), 5-hydroxy-7-methoxyflavone (6), 5-hydroxy-7,4′-dimethoxyflavone (7), 5-hydroxy-3,7-dimethoxyflavone (8), and 5-hydroxy-3,7,4′-trimethoxyflavone (9) elucidated by nuclear magnetic resonance spectroscopy; (**D**) IL-6 concentration (pg/mL) in the culture supernatants of HeLa cells treated with different concentrations of KP extract (0–15 µg/mL) for 24 h as measured by enzyme-linked immunosorbent assay (ELISA). The maximum concentration of dimethyl sulfoxide (DMSO) at 0.02% was used as a vehicle control. Data represent mean ± SD of three independent experiments. * *p* < 0.05. EGF, epidermal growth factor.

The major chemical constituents of the ethanolic extract from *Kaempferia parviflora* were determined by high performance liquid chromatograph (HPLC) in comparison with nine standard compounds. The chromatogram of KP extract was identified by comparing their retention times to those of the standard methoxyflavones (Figure 1A,B). The results indicated that KP ethanolic extract contains methoxyflavones as major compounds, which are 3,5,7,3′,4′-pentamethoxyflavone (1), 5,7,4′-trimethoxyflavone (2), 3,5,7-trimethoxyflavone (3), 3,5,7,4′-tetramethoxyflavone (4), 5-hydroxy-3,7,3′,4′-tetramethoxyflavone (5), 5-hydroxy-7-methoxyflavone (6), 5-hydroxy-7,4′-dimethoxyflavone (7), 5-hydroxy-3,7-dimethoxyflavone (8), and 5-hydroxy-3,7,4′-trimethoxyflavone (9). The structures of these nine standard compounds are shown in Figure 1C. 

We next performed an MTT assay to determine the effects of KP extract on cell viability (with or without the presence of EGF). We found that the viability of HeLa cells was decreased when the concentration of KP extract was increased (both without or with the presence of EGF). Therefore, the obtained results exhibited two different ranges of concentration, which are non-cytotoxic and cytotoxic concentration. Data for the MTT viability assay are presented in  Figure S1. As we were particularly interested in studying the effects of KP on modulating signal transduction pathways in living cancer cells and based on the fact that cell death can affect the intracellular protein content, we thus selected three different noncytotoxic concentrations (3.75, 7.5, and 15 µg/mL) for all experiments to ensure that the inhibitory effects of KP are not primarily caused by the activation of cell death, which results in protein deconstruction. 

IL-6 has been reported to be highly expressed in invasive cervical carcinoma and is associated with the pathogenesis of HPV-related cervical carcinoma [2,30,31]. To determine whether KP can suppress the secretion of IL-6 by HPV18-positive HeLa cells in response to EGF stimulation, we performed enzyme-linked immunosorbent assay (ELISA) to measure the level of IL-6 in the culture supernatants of HeLa cells either untreated, treated with EGF, or treated with EGF and KP at different nontoxic concentrations for 24 h. The results showed that the basal level of IL-6 in the culture supernatant of the untreated cells was 356.2 ± 19.97 pg/mL (Figure 1D). The addition of EGF to HeLa cells for 24 h resulted in a significant increase in IL-6 secretion in the supernatant to 426.8 ± 12.40 pg/mL (*p* = 0.0011), meaning that EGF-induced production of IL-6 was increased around 20%, as compared with that of the untreated cells. Interestingly, KP extract showed an inhibitory effect on IL-6 production in response to the influence of EGF, and the reduction of the cytokine was observed to be in a concentration-dependent manner. Specifically, KP extract at 7.5 and 15 µg/mL could significantly reduce IL-6 secretion to 326.8 ± 18.19 and 242.6 ± 13.85 pg/mL, respectively (*p* < 0.0001), which were both less than the basal level of IL-6 from untreated cells. The dimethyl sulfoxide (DMSO) vehicle control (0.02%) showed no inhibitory effect on IL-6 secretion (Figure 1D). These results suggest that KP, containing methoxyflavones, could be able to suppress the production and secretion of IL-6 from HeLa cervical cells stimulated with EGF.

### 2.2. KP Inhibits EGF-Induced STAT3 Activation

**Figure 2:**
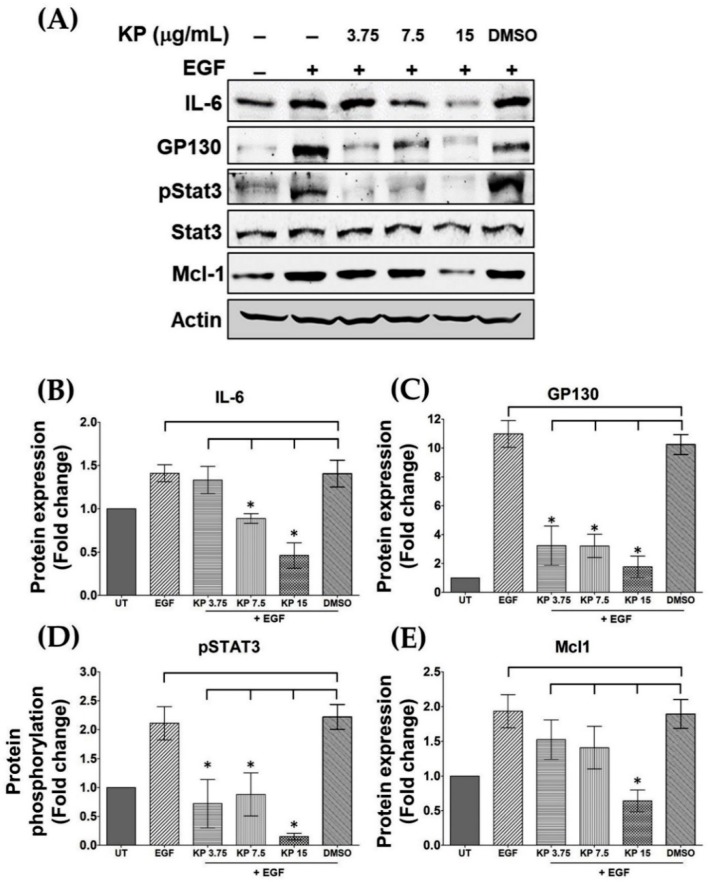
(**A**) The effects of KP on suppressing EGF-induced expression of intracellular signaling proteins determined by Western blot analysis of cell lysates from HeLa cells exposed to KP extract at different concentrations; (**B**) quantitative analysis of cellular level of interleukin (IL)-6 protein; (**C**) quantitative analysis of cellular level of GP130 protein; (**D**) quantitative analysis of signal transducers and activators of transcription 3 (STAT3) phosphorylation; (**E**) quantitative analysis of cellular level of Mcl-1 protein. The protein expression levels of IL-6, GP130, and Mcl1 were normalized against actin, while the phosphorylated form of STAT3 was normalized against total STAT3 protein. Data represent mean ± SD of three independent experiments. * *p* < 0.05 in comparison with cells treated with DMSO + EGF.

On the basis of our observation from ELISA that KP could potently suppress IL-6 levels in the culture supernatants of HeLa cells stimulated with EGF, we tested whether the intracellular production of IL-6 is affected by the action of KP. We thus performed Western blot analysis to detect total IL-6 protein in the cell lysates. The results showed that HeLa cells without any treatment expressed cellular IL-6 (Figure 2A), and the cells responded to EGF stimulation by increasing production of cellular IL-6 by 1.41 ± 0.09 fold. However, in comparison with HeLa cells treated with DMSO in combination with EGF, KP extract at 7.5 and 15 µg/mL could significantly reduce cellular IL-6 production to 0.80 ± 0.04 fold, respectively, whereas DMSO did not have any effect on suppressing EGF-induced cellular IL-6 expression (Figure 2A,B).

Because we observed that KP could suppress the influence of EGF on inducing the upregulation of both intracellular and secreted IL-6 in HeLa cells, we further defined whether KP has the ability to regulate the production of other molecular players in IL-6 signaling. We examined GP130, which is one of the earliest molecules responsible for conveying the signal transduction upon IL-6 binding. We demonstrated that the untreated HeLa cells expressed a low basal level of GP130 protein, but EGF could significantly increase GP130 protein production to approximately 10-fold (10.96 ± 0.92 fold; *p* < 0.0001). Surprisingly, KP strongly reduced EGF-induced expression of this protein at all tested concentrations (3.75, 7.5, and 15 µg/mL) to 3.23 ± 0.95 (*p* = 0.0228), 3.21 ± 0.57 (*p* = 0.0113), and 1.76 ± 0.53 (*p* = 0.0072) fold, respectively, compared with that of cells treated with DMSO + EGF (Figure 2A,C). Additionally, we analyzed another key signaling molecule, STAT3, and found that neither EGF nor KP extract had effects on STAT3 protein levels. Nevertheless, EGF noticeably induced phosphorylation of STAT3 by 2-fold, and KP at all selected concentrations almost completely inhibited EGF-induced phosphorylation of STAT3 with the maximum inhibition observed in cells treated with KP at 15 µg/mL (0.15 ± 0.03 fold; *p* < 0.0001) in comparison with that of cells treated with DMSO + EGF (Figure 2A,D). Additionally, KP showed strong inhibitory effects over the influence of EGF on the expression level of the anti-apoptotic protein Mcl-1 (Figure 2A,E). These data suggest that KP can negatively regulate the expression level and the phosphorylation of important molecular players in the IL-6/STAT3 signal transduction pathway in HeLa cells.

### 2.3. EGF has no Effects on Nuclear Factor Kappa B (NF-κB) Activation in HeLa Cells, and the Suppression of IL-6 Production by KP is not Regulated through NF-κB Inhibition

**Figure 3:**
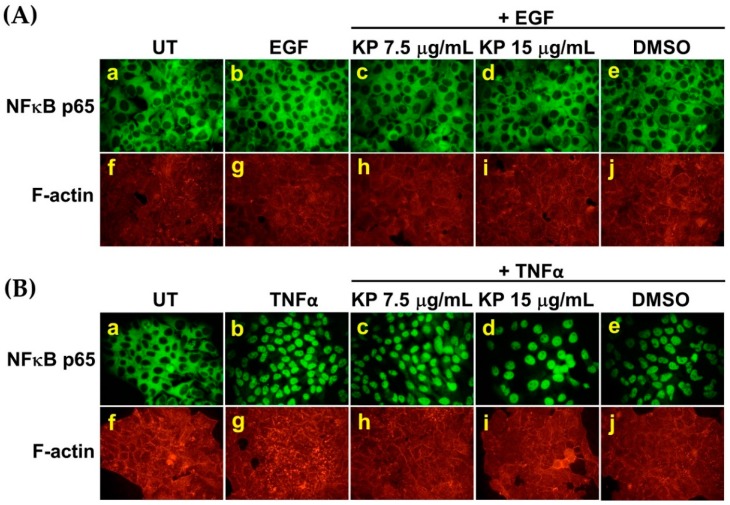
The effects of KP on nuclear localization of nuclear factor kappa B (NF-κB) in HeLa cells. (**A**) Representative images from immunofluorescence study showing NF-κB staining (green) in HeLa cells with or without treatment with KP at 7.5 and 15 µg/mL or DMSO for 24 h and stimulated with EGF for 15 min; (**B**) representative images from an immunofluorescence study showing NF-κB staining (green) in HeLa cells with or without treatment with KP at 7.5 and 15 µg/mL or DMSO for 24 h and stimulated with tumor necrosis factor-alpha (TNF-α) for 15 min. Filamentous actin [F-actin (red)] was stained with Phalloidin-TRITC. Micrographs were captured at 40× magnification. Data are representatives of three replicates.

It has been reported that the activation of EGFR by EGF in some cancer cell lines results in NF-κB activation. Therefore, to address our hypothesis that EGF may increase the production and secretion of IL-6 in HeLa cells through activating NF-κB, and KP may suppress this effect of EGF, we performed immunofluorescence to detect changes in NF-κB p65 localization after EGF stimulation. NF-κB p65 was detected in the cytoplasm of untreated cells (Figure 3A(a)). Surprisingly, EGF treatment for 15 min did not induce NF-κB p65 nuclear translocation in HeLa cervical cancer cells (Figure 3A(b)). We further incubated cells with EGF for 24 h, but the results were the same, demonstrating that EGF did not induce NF-κB p65 nuclear translocation in HeLa cells (Figure S2). Additionally, HeLa cells treated with KP extract at different concentrations in the presence of EGF showed no difference in NF-κB p65 localization pattern, as compared with cells in the untreated, EGF-treated, and DMSO-treated groups (Figure 3A(c–e)). We further verified NF-κB p65 nuclear translocation by inducing cells with 20 ng/mL of tumor necrosis factor-alpha (TNF-α) for 15 min. The results clearly showed that TNF-α potently induced NF-κB p65 translocation into the nucleus of HeLa cells (Figure 3B(b)) when compared with the cytoplasmic NF-κB localization in untreated cells (Figure 3B(a)). Similarly, KP extracts did not inhibit TNF-α-induced NF-κB p65 nuclear translocation (Figure 3B(c–e)). In addition to NF-κB p65, HeLa cells in all groups were co-stained with Phalloidin-TRITC for visualizing filamentous actin, and the signals in all groups were approximately equal (Figure 3A(f–j) and Figure 3B(f–j)). Our findings clearly indicate that EGF does not induce NF-κB activation, and KP does not have any effect on NF-κB nuclear localization status, which suggests that KP suppresses IL-6 production via targeting other signaling molecules. 

### 2.4. KP Inhibits EGF-Induced Activation of EGFR

**Figure 4:**
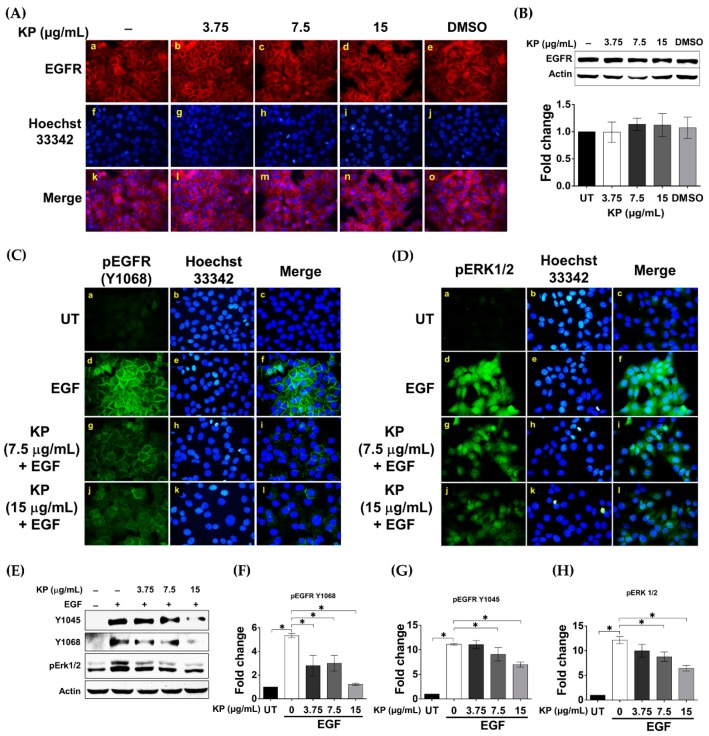
(**A**) The effects of KP on EGF receptor (EGFR) expression in HeLa cells treated with different concentrations of KP or DMSO for 24 h; (**B**) Western blotting with quantitative analysis of total EGFR in HeLa cells treated with different concentrations of KP or DMSO for 24 h; (**C**) immunofluorescence for EGFR phosphorylation (pEGFR) at tyrosine 1068 (Y1068) (green) in HeLa cells treated with different concentrations of KP for 24 h and stimulated with EGF for 2 min; (**D**) immunofluorescence for ERK1/2 phosphorylation (green) in HeLa cells treated with different concentrations of KP for 24 h and stimulated with EGF for 15 min. Nuclei were counterstained with Hoechst 33342 (blue); (**E**) Western blot analysis for EGFR phosphorylation at Y1045 and Y1068 and pERK1/2 in HeLa cells treated with different concentrations of KP for 24 h and stimulated with EGF for 2 min; (**F**) quantitative analysis for EGFR phosphorylation at Y1068; (**G**) quantitative analysis for EGFR phosphorylation at 1045; (**H**) quantitative analysis for ERK1/2 phosphorylation. Micrographs were captured at 40× magnification. Data represent mean ± SD of three replicates. * *p* < 0.05.

It is well known that EGFR can activate STAT3 phosphorylation. Consistently, our data (shown in Figure 2) depicted the potent ability of KP in inhibiting EGF-induced phosphorylation of STAT3. Therefore, the effects of KP on suppressing the upstream EGFR signal transduction pathways were determined. One possible mechanism by which KP could inhibit the EGFR signal transduction pathway is through downregulation of EGFR protein expression. To test this hypothesis, we performed an immunofluorescence study to visualize the expression of EGFR on the surface of HeLa cells treated with KP extract at different concentrations. We found that the signal intensity of EGFR in HeLa cells treated with KP (at all concentrations) for 24 h (Figure 4A(b–d)) was not different from that seen in the untreated (Figure 4A(a)) and DMSO-treated cells (Figure 4A(e)). Supporting this, the results from the Western blot analysis confirmed that KP extract did not significantly affect the expression levels of cellular EGFR protein (Figure 4B). We further tested our second hypothesis that KP may be able to suppress the activation of EGFR upon EGF stimulation. The results from an immunofluorescence study clearly showed the potent inhibitory effect of KP on EGF-stimulated EGFR activation. In particular, EGF addition markedly activated EGFR phosphorylation at tyrosine 1068 residue (Y1068) (Figure 4C(d–f)) and ERK1/2 phosphorylation (Figure 4D(d–f)). However, KP at 7.5 and 15 µg/mL dramatically reduced EGF-induced stimulation of EGFR and ERK phosphorylation (Figure 4C(g–l) and Figure 4D(g–l), respectively). Consistently, results from the Western blot analysis verified that the presence of KP inhibited EGFR activation upon EGF stimulation. In addition to the phosphorylation of Y1068 (Figure 4E,F), the phosphorylation status of EGFR at tyrosine 1045 (Y1045) was also shown to be decreased in cells treated with KP extract and stimulated with EGF (Figure 4E,G). Moreover, the phosphorylation of ERK1/2 was also confirmed to be reduced by the action of KP in a concentration-dependent manner (Figure 4E,H). All of these results suggest that KP can suppress EGF-stimulated EGFR activation, which may result in downregulation of IL-6 production in HeLa cells.

### 2.5. KP Exhibits Anti-Proliferative Effects over EGF Stimulation

**Figure 5:**
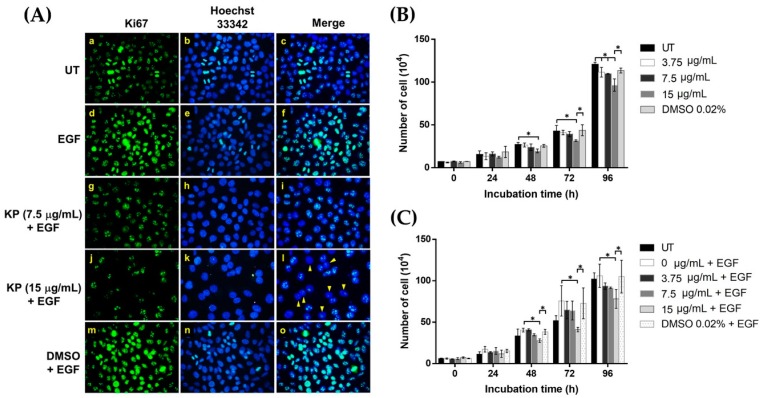
The effects of KP on reducing the proliferative influence of EGF in HeLa cells. (**A**) Immunofluorescence study detecting the proliferative marker, Ki-67, protein (green) in the nuclei of HeLa cells treated with different concentration of KP or DMSO, with or without the presence of EGF for 24 h. Nuclei were counterstained with Hoechst 33342 (blue). Arrow heads (yellow) indicate the nuclei of cells with very low or undetectable signal of Ki67 protein; (**B**) the number of HeLa cells treated with non-toxic concentrations of KP (3.75, 7.5, and 15 μg/mL) without the presence of EGF (0–96 h); (**C**) the number of KP-treated HeLa cells with the presence of EGF (0–96 h). Micrographs were captured at 40× magnification. Data represent mean ± SD of three replicates. * *p* < 0.05.

On the basis of the fact that EGF stimulates cell growth and survival and is a regulator of cancer cell proliferation, we investigated the expression of Ki-67, a marker for cell proliferation that has been used as a potential prognostic or predictive marker in several malignant tumors. To determine whether KP can reduce EGF-induced cell proliferation, untreated and KP-treated HeLa cells were stained to detect Ki-67 expression. The results showed that all of the untreated cells exhibited a high basal level of Ki-67 protein in the nuclei of HeLa cells, and many cells were observed to be in the late mitotic phase, where the duplication of the nuclei was clearly seen (Figure 5A(a–c)). We observed that during mitotic cell division, the intensity of Ki-67 signal was markedly increased. When EGF was present, all nuclei of HeLa cells showed a remarkable increase in Ki-67 expression, with many mitotic cells detected (Figure 5A(d–f)). Interestingly, when HeLa cells were incubated with EGF in the presence of KP extract at 7.5 µg/mL, we observed a drastic decrease in Ki-67 intensity (Figure 5A(g–i)). When the concentration of KP extract was increased to 15 µg/mL, a substantial reduction in Ki-67 expression was clearly seen (Figure 5A(j–l)). We also observed that some cell nuclei exhibited a very low or undetectable level of Ki-67 (Figure 5A(l), arrow heads). HeLa cells treated with DMSO vehicle plus EGF revealed similar characteristics to those cells in the EGF-treated group (Figure 5A(m–o)). We further performed cell counting at 24, 48, 73, and 96 h after KP treatment and found that KP extract at 15 μg/mL significantly reduced the number of cells at 48 h and 72 h, whereas KP at all concentrations causes the reduction of cell number in a concentration-dependent manner at 96 h (Figure 5B). The inhibitory effects of KP were still seen in the treatment with the presence of EGF, where KP at 15 μg/mL significantly reduced the HeLa cancer cell number at 48, 72, and 96 h (Figure 5C). These data confirm the anti-proliferative effects of KP against EGF-stimulated HeLa cells.

### 2.6. KP Inhibits IL-6-Induced STAT3 Activation and Expression of the Anti-Apoptotic Protein Mcl-1

**Figure 6:**
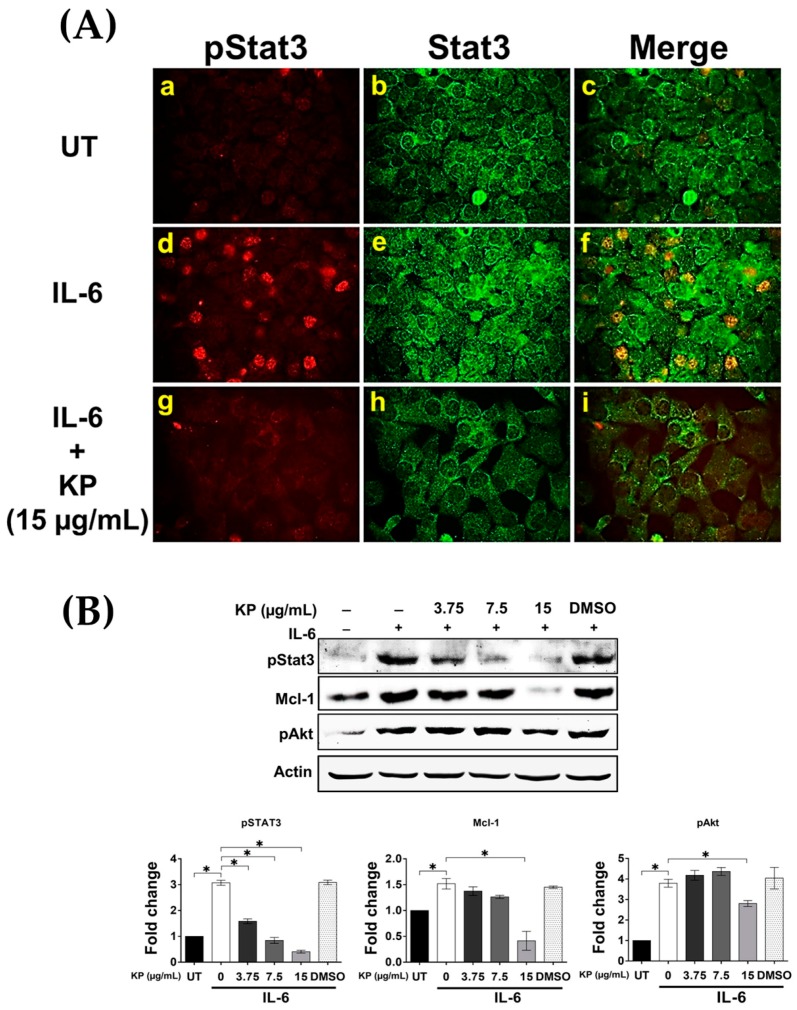
The effects of KP on suppressing IL-6-induced STAT3 signaling in HeLa cells. (**A**) Immunofluorescence for nuclear STAT3 phosphorylation (red) in HeLa cells treated with KP at 15 μg/mL, with the presence of human recombinant IL-6 (50 ng/mL) for 24 h. The total STAT3 protein (green) was detected to visualize total expression of STAT3; (**B**) Western blotting and quantitative analysis for pSTAT3, Mcl-1, and pAkt in HeLa cells treated with different concentrations of KP or DMSO, with the presence of human recombinant IL-6 for 24 h. Micrographs were captured at 40× magnification. Data represent mean ± SD of three replicates. * *p* < 0.05.

Because we observed that KP could significantly reduce the production and secretion of IL-6, as well as the level of GP130 in HeLa cells stimulated with EGF, we further explored whether KP has the ability to suppress IL-6 signaling. On the basis of the fact that classic IL-6 signaling requires binding of IL-6 to the interleukin-6 receptor (IL-6R) and subsequent activation of STAT3, we performed an immunofluorescence study to determine the effects of KP on STAT3 activation in response to exogenous IL-6 treatment. The results showed that stimulation of HeLa cells with 50 ng/mL of human recombinant IL-6 led to phosphorylation of STAT3 in the nucleus of many cells (Figure 6A(d–f)), but this effect of IL-6 on inducing STAT3 phosphorylation was almost completely inhibited by KP extract at 15 µg/mL (Figure 6A(g–i)). We further verified the inhibitory effects of KP by Western blot analysis, and the results showed that IL-6 at 50 ng/mL induced strong phosphorylation of STAT3 (3.07 ± 0.09 fold; *p* < 0.0001) (Figure 6B) in the nuclei of HeLa cells. As expected, we observed that KP extract at 3.75, 7.5, and 15 µg/mL significantly suppressed IL-6-induced STAT3 phosphorylation to 1.58 ± 0.09, 0.84 ± 0.11, and 0.40 ± 0.05 fold, respectively, (*p* < 0.0001) (Figure 6B). Additionally, KP at 15 µg/mL showed significant inhibitory effects over the influence of EGF on the phosphorylation of Akt and the expression level of the anti-apoptotic protein, Mcl-1 (Figure 6B). These results indicate that KP potently inhibits IL-6-induce STAT3 activation and the expression of an anti-apoptotic protein McL-1, suggesting that KP may increase the sensitivity of HPV18 positive cervical cancer cells to cytotoxic standard chemotherapy drugs.

## 3. Discussion

One of the most common neoplastic diseases that affects women worldwide is cervical cancer. This type of cancer has been proven to be associated with human papillomavirus (HPV) infection [32]. The local immune system of the genital tract is assumed to be responsible for defending against HPV infection and controlling HPV-related cervical cancer. However, cervical cancer development and progression may be related to a persistent immune response and inflammation. This statement can be supported by many epidemiologic studies reporting a strong correlation between chronic inflammation resulting from multiple sexually transmitted pathogens and the development of cervical cancer [33]. IL-6 has been proved to be involved in the proliferation and differentiation of various malignant tumors [34,35]. Considering the link between inflammatory cytokine production and cervical cancer, various cytokines have been implicated in the pathogenesis of cervical cancer, among which IL-6 has received particular attention. An example of this link is shown by a study reporting that consistently higher expression of IL-6 and VEGF is found in cancerous tissues than in the adjacent noncancerous tissues in early-stage cervical cancer patients [36]. Another study by the same research group demonstrated that cervical cancer cells increase IL-6 production, and the overexpression of this cytokine may promote cervical tumorigenesis by activating VEGF-mediated angiogenesis via a STAT3 pathway [37]. There have been attempts to understand the mechanism of IL-6 in promoting cervical cancer development. One study has uncovered that E6 protein produced from HPV 16/18 activates IL-6/STAT3 signaling, which further induces the senescence of cancer-associated fibroblasts, which may remodel the tumor microenvironment to promote cervical epithelial cells from a chronic tumor-prone inflammatory state to malignant proliferation [38]. On the basis of high microenvironmental IL-6 levels promoting cervical cancer development and angiogenesis, inhibition of the biological activity of IL-6 may be potentially beneficial for the treatment of cervical cancer.

We first determined the basal level of IL-6 produced by HeLa cells and found that HeLa cells secreted a high basal level of IL-6 in the serum-free culture supernatant collected after 24 h. The explanation for this phenomenon may be because of the fact that HeLa cervical cancer cells contain HPV-18 sequence, which stimulates constitutive expression of IL-6. Besides cervical cancer cells, the effect of HPV16/18 E6 and E7 on upregulating IL-6 expression has been reported in E6- and E7-transfected A549 cells, which are a non-small cell lung cancer cell line [39]. In addition to viral infection, EGFR has been documented to play a role in increasing IL-6 expression. For example, EGFR activation in response to EGF binding mediates IL-6 production in ovarian and lung cancer cells [40,41]. Moreover, the production of IL-6 has been reported to be regulated at the transcriptional level by the MAPK signal transduction pathway in HeLa cells induced with IL-1β [42]. Therefore, it is possible that EGF can also regulate the production of IL-6 in HPV18 positive cervical cancer cells through induction of similar signaling pathways. The results from our current study showed that the addition of EGF enhanced IL-6 secretion in the culture supernatant of HeLa cells, and that KP extract could significantly reduce the effect of EGF in inducing IL-6 secretion in a concentration-dependent manner. In particular, KP extract at the maximum concentration exhibited a potent inhibitory activity in which IL-6 secretion was reduced to a level lower than that of the untreated cells (without the presence of EGF). Similar results were observed when we lysed HeLa cells and detected the intracellular level of IL-6 protein. These results indicate that KP can interfere with EGFR signaling in stimulating the production and secretion of IL-6, at least in part through inhibiting MAPK activation. Our previous report supports this statement because we clearly demonstrated that KP could potently suppress EGF-induced ERK1/2 and PI3K/Akt phosphorylation in HeLa cervical cancer cells and SKOV-3 ovarian cancer cells [29,43].

On the basis of the observation that EGF could increase IL-6 production in HeLa cells, it is reasonable to hypothesize that EGF may also induce the expression of other important molecular players in the IL-6/STAT3 signal transduction pathway in HeLa cells. As expected, EGF stimulation for 24 h strongly induced the expression of GP130 protein, which is a signal-transducing subunit shared by the receptors for the IL-6 family of cytokines [44,45,46]. Surprisingly, KP at all tested concentrations was able to suppress EGF-induced GP130 production. These results indicate that KP can mediate the downregulation of GP130, which is important for autocrine signaling in response to IL-6 binding. EGFR is well known to catalyze the phosphorylation of STAT3 in response to EGF [47], and upon activation by EGF, EGFR dimerizes to facilitate cross-phosphorylation of several tyrosine residues, including tyrosine 1068 (Y1068), which is the binding site for STAT3 [48]. We thus examined the effects of KP on suppressing EGF-induced STAT3 activation. We discovered that EGF could induce the phosphorylation of STAT3 in HeLa cervical cancer cells. Our data are in line with previous reports revealing that EGF can cross talk with IL-6/STAT3 signaling in other cell types. For instance, one study showed that STAT3 phosphorylation in response to IL-6 is prolonged by EGF addition [22]. However, HeLa cells stimulated with EGF in the presence of KP showed a drastic reduction in STAT3 phosphorylation. Together, our data confirmed that KP can inhibit IL-6 production and STAT3 activation stimulated by EGF. We also tested the expression level of the anti-apoptotic protein, Mcl-1, which has been reported to be concomitantly expressed with IL-6 in human invasive cervical carcinomas, and Mcl-1 expression has been shown to be induced by IL-6 through the PI3K/Akt pathway [10]. Mcl-1 can be upregulated by trophic factor cytokines including IL-6 [49] and growth factors including EGF [50,51]. Specifically, EGF activates Mcl-1 translation through the RAS–RAF–MEK–ERK and ELK1 pathway. Interestingly, our results demonstrated that KP markedly reduced EGF-induced Mcl-1 production in HeLa cells, confirming its anti-cancer ability to modulate the EGFR/MAPK signaling cascade.

The inducible nuclear factor kappa B (NF-κB) regulates the expression of a wide variety of genes during inflammatory responses and the initiation and progression of cancer [52,53]. Interestingly, EGF has been demonstrated to stimulate IKK-dependent NF-κB activation via PI3K and protein kinase C, resulting in cell-cycle progression in estrogen-receptor negative breast cancer cells [54]. Specifically, it is well known that NF-kB regulates Mcl-1 and IL-6 expression, and these two proteins are also highly expressed in invasive cervical cancer, but not in normal cervical tissues [10]. On the basis of these previous reports, NF-kB may be involved in EGF-induced IL-6 production in HeLa cervical cancer cells. Therefore, we explored whether KP suppresses EGF-induced production of IL-6 by blocking NF-kB activation. We monitored the activation of NF-kB by performing an immunofluorescence study to visualize its nuclear translocation in response to EGF stimulation. Surprisingly, EGF did not stimulate NF-kB nuclear translocation at any time points in this cell line. Unsurprisingly, KP was observed to have no effect on the localization of NF-kB. To confirm that HeLa cells used in our study remain responsive to inflammatory stimuli, we stimulated the cells with tumor necrosis factor alpha (TNFα) and found that HeLa cells rapidly responded to TNFα by activating NF-kB nuclear translocation, indicating that the NF-kB signaling pathway is functional in this cell line. However, KP failed to inhibit TNFα-stimulated NF-kB activation. These data indicate that EGF does not play a major role in activating NF-kB to upregulate IL-6, and we could rule out the possibility that KP suppresses the production and secretion of IL-6 in HeLa cells through modulation of the NF-kB signaling pathway. We continued our attempts to understand how KP inhibits EGF-induced IL-6 production in HeLa cells by focusing on the ability of KP to suppress the upstream events of EGFR signaling. One possibility is that KP may downregulate the expression of EGFR. Therefore, we evaluated the expression of EGFR by staining to detect its presence on the surface, as well as by Western blot analysis. However, we observed no significant difference in the level of EGFR, indicating that KP does not suppress the EGF-induced IL-6 production by decreasing the expression of EGFR. As we previously reported that KP suppresses ERK1/2 and Akt phosphorylation in response to EGF stimulation in HeLa cells [29], it is possible that KP may suppress the activation of EGFR upon EGF binding. As expected, data from an immunofluorescence study showed that KP dramatically inhibited tyrosine phosphorylation at residue 1068 of EGFR, which is a binding site for the GRB2 adaptor protein [55] and STAT3 [48]. The phosphorylation of ERK1/2 was verified to be inhibited by KP in a concentration-dependent manner. The results showed that the inhibitory effects of KP occur at the upstream events of the MAPK signaling pathway. We verified the immunofluorescence results by Western blot analysis and found similar results, whereby KP suppressed phosphorylation of EGFR at both tyrosine 1068 and tyrosine 1045. These results clearly indicate that KP is able to inhibit the activation of EGFR in response to EGF stimulation, which explains why KP can suppress EGF-induced IL-6 production and secretion in HeLa cells. In addition, we designed an experiment to confirm the anti-proliferative effects of KP against EGF stimulation by visualizing the presence of Ki-67 protein in the nuclei of HeLa cells. Ki-67 is a nuclear nonhistone protein [56] that is universally expressed in proliferating cells. Research studies have explored the use of Ki-67, along with other markers, as a potential prognostic or predictive markers in many malignant diseases [57]. As expected, we found that KP reduced the expression of Ki-67 in the nuclei of HeLa cells, with a very low number of mitotic cells detected, whereas EGF-treated cells exhibited high expression of nuclear Ki-67 and a high number of dividing cells. Moreover, we performed additional experiment to evaluate a growth curve of HeLa cells treated with KP with or without the presence of EGF. Data confirmed that KP significantly reduces the cell number over 96 h even when EGF was present. These data verify that KP has effects on HeLa cell proliferation. Consistently, the results support our previous study, wherein we discovered that KP significantly increases the time for cancer cells to migrate to the scratched site, as examined by wound healing assay [29,43]. Altogether, these results strongly suggest that KP suppresses EGF-induced IL-6 production through inhibiting the activation of EGFR and its subsequent proliferative effects. It would significantly help our understanding of the molecular mechanism of action of KP if we knew exactly where the active methoxyflavones of KP interacts with EGFR to abolish receptor activation. The current study did not provide any information on how KP interrupts EGFR signaling at the structural level, as the KP crude extract contains mixed potential active methoxyflavones. However, on the basis of our data, it is possible that KP methoxyflavones may bind to certain domains of EGFR and interfere with the receptor autophosphorylation. This hypothesis can be tested in the future with computational approaches to analyze compound–protein association at the structural and energetic level together with functional tests.

Considering the fact that higher expression of IL-6 is evident in cancerous tissues than in adjacent noncancerous tissues in early-stage cervical cancer patients [36], and our observation that KP decreases EGF-induced IL-6 production and secretion, it is reasonable to imagine that KP may hamper cancer development. One possible hypothesis is that KP may be able to interrupt the IL-6 autocrine machinery. Therefore, we examined whether KP inhibits the effects of exogenous IL-6 on conveying the signal through its receptors. On the basis of one of our interesting observations demonstrating that KP strongly inhibited EGF-induced upregulation of GP130, we anticipated that the reduction of this cytokine receptor subunit would make HeLa cells less responsive to extracellular IL-6. As anticipated, our results clearly showed that KP totally inhibited STAT3 phosphorylation in the nuclei of cells stimulated with human recombinant IL-6. Consistently, we noticed that KP also suppressed Akt phosphorylation upon IL-6 stimulation. On the basis of the previous report that IL-6 induces the expression of the anti-apoptotic protein Mcl-1 in cervical cancer cells through a PI3K/Akt-dependent pathway [10], we tested the expression level of Mcl-1 protein and found that KP potently suppressed the IL-6-induced expression of this anti-apoptotic protein in HeLa cells. 

## 4. Materials and Methods 

### 4.1. Plant Material and Extraction of Kaempferia parviflora Rhizomes

The preparation of the plant extract was performed exactly as reported in our previous study [29]. Briefly, rhizomes of KP harvested from the CMU-RSPG Kaempferia housing at Chiang Dao, Chiang Mai Province, Thailand with voucher specimen number, R-CMUKP002, were weighed, chopped, and extracted with 95% ethanol (Merck KGaA, Darmstadt, Germany) at room temperature (RT) for three days. The ethanolic extract was filtered; concentrated using a rotary evaporator; lyophilized; and kept in an air-tight, light protected container. The KP extract stock solution was freshly prepared using DMSO (Sigma-Aldrich, Saint Louis, MO, USA) prior to each assay. One gram of ethanolic KP crude extract was dissolved in 1 mL of 100% DMSO to make a stock solution of 1 g/mL, and the stock was pre-diluted in medium prior to each treatment. Each experiment was performed with three independent batches of KP extract, each assayed in triplicate. The final concentration of DMSO was maintained below 0.5% v/v throughout all experiments.

### 4.2. High Performance Liquid Chromatograph Analysis

The presence of major methoxyflavones in the KP ethanolic extract was identified by high performance liquid chromatograph (HPLC) compared with standard compounds. Nine standard compounds, including 3,5,7,3′,4′-pentamethoxyflavone (1), 5,7,4′-trimethoxyflavone (2), 3,5,7-trimethoxyflavone (3), 3,5,7,4′-tetramethoxyflavone (4), 5-hydroxy-3,7,3′,4′-tetramethoxyflavone (5), 5-hydroxy-7-methoxyflavone (6), 5-hydroxy-7,4′-dimethoxyflavone (7), 5-hydroxy-3,7-dimethoxyflavone (8), and 5-hydroxy-3,7,4′-trimethoxyflavone (9), were obtained from the Eco-friendly Product Research laboratory (EfPRL), Department of Chemistry, Faculty of Science, Chiang Mai University, Thailand. The structures of these standard compounds were elucidated using ^1^H Nuclear Magnetic Resonance Spectroscopy, BrÜker DPX 400 spectrometer, (Bruker BioSpin Corporation, Billerica, MA, USA) and confirmed by comparing to the previous literature that identified these compounds in KP [58,59]. HPLC analysis for methoxyflavones in KP extract was performed on an Agilent 1100 series (Agilent Technologies, Santa Clara, CA, USA) equipped with a UV/vis detector. The separation was carried out on a C18 column (250 mm × 4.6 mm, internal diameter 5 µm, Hypersil™, Sigma-Aldrich, Saint Louis, MO, USA). The mobile phase was methanol/0.5% acetic acid (65:35, v/v), operation at a flow rate of 1.0 mL/min. The injection volume was set as 20 µL.

### 4.3. Cell Culture

The human HeLa cell line [HeLa 229 (ATCC^®^ CCL-2.1^TM^)] used in this study was obtained from ATCC (ATCC, Manassas, VA, USA). The cells were cultured in complete medium, which is Dulbecco’s modified Eagle’s medium (DMEM) (Gibco, Thermo Fisher Scientific, Waltham, MA, USA), supplemented with 10% fetal bovine serum (Merck KGaA, Darmstadt, Germany) and antibiotics (100 U/mL penicillin and 100 μg/mL streptomycin) (Gibco, Thermo Fisher Scientific, Waltham, MA, USA), and maintained under a humidified atmosphere of 37 °C, 5% CO_2_. The cells were sub-cultured every 2–3 days. 

### 4.4. Cell Viability Assay

The effect of KP on cell viability was evaluated using 3-(4,5-dimethylthiazol-2-yl)-2,5-diphenyltetrazolium bromide (MTT) (Sigma-Aldrich, Saint Louis, MO, USA) to obtain the range of toxic and nontoxic concentrations. The MTT assay was performed according to a previously published protocol [60]. HeLa cells were seeded in 96-well plates at a density of 1 × 10^4^ cells per well for 24 h in complete medium. Cells were then treated with KP extract at various concentrations (0–1 mg/mL) or with vehicle (DMSO at 0.001%–0.1%) for 48 h, after which cells were exposed to the MTT reagent (0.5 mg/mL in PBS) for 2 h at 37 °C, 5% CO_2_. After aspirating the culture supernatants, 200 μL of DMSO was added to each well, and the plates were incubated in the dark for 10 min. The absorbance at 590 nm was measured using a microplate reader (BioTek Instruments, Winooski, VT, USA). The cell viability assay was performed three times, and each assay was undertaken in triplicate (n = 9 in three individual experiments). Data for MTT viability assay are presented in  Figure S1. Three different noncytotoxic concentrations (3.75, 7.5, and 15 µg/mL) were selected for all experiments.

### 4.5. Enzyme-Linked Immunosorbent Assay (ELISA)

HeLa cells were left untreated or treated with different nontoxic concentrations (0–15 μg/mL) of KP with the presence of 100 ng/mL of recombinant human epidermal growth factor (EGF) (Immuno Tools, Friesoythe, Germany) for 24 h. The supernatants of the HeLa cells were harvested and kept at 4 °C until use. The concentration of IL-6 in the culture supernatants was measured by using ELISA MAX™ Deluxe Set Human IL-6 (BioLegend, San Diego, CA, USA) following the manufacturer’s instruction. Briefly, the capture antibody diluted in a coating buffer was added into individual wells of a microtiter plate and incubated at 4 °C overnight. Then, the plate was added with blocking buffer for 1 h, at room temperature (RT). Sample supernatants were added into each well and plates were incubated at RT for 2 h. After washing, a detection antibody solution was added to each well for 1 h. Then, the plate was washed four times before adding a diluted avidin–HRP solution and incubating for 30 min. The signal was developed by the addition of freshly mixed TMB substrate solution. A stop solution was added to each well, and the absorbance was read at 450 nm and 570 nm with a microplate reader (BioTek Instruments, Winooski, VT, USA).

### 4.6. Western Blot Analysis

For studying the effects of KP on inhibiting EGF-induced expression and phosphorylation of important proteins in IL-6/STAT3 signaling pathway, HeLa cells at 90% confluence cultured in 3 cm dishes were treated with KP extract at different concentrations (0–15 μg/mL) in serum-free media with the presence of 100 ng/mL of EGF for 24 h. For studying the effects of KP on total EGFR protein expression, HeLa cells were treated with KP extract at different concentrations (0–15 μg/mL) in serum-free medium (without the presence of any growth factors or cytokines) for 24 h. For studying the effects of KP on suppressing EGFR phosphorylation, HeLa cells were treated with KP at different concentrations (0–15 μg/mL) for 24 h and activated with 100 ng/mL EGF for 2 min before harvesting. For studying the effects of KP on inhibiting IL-6-induced STAT3 signaling, HeLa cells were treated with KP at different concentrations (0–15 μg/mL) in serum-free media in the presence of 50 ng/mL of human recombinant IL-6 for 24 h. After treatment, the culture supernatants were discarded, cells were rinsed with PBS, and cell lysates were prepared by adding 300 μL of 1x reducing Laemmli buffer into the sample dishes. Samples were collected, heated at 95°C for 5 min, separated by SDS-PAGE, and electroblotted onto PVDF membranes (GE Healthcare Life Sciences, Marlborough, MA, USA). Membranes were blocked with 5% BSA (Merck KGaA, Darmstadt, Germany) in TBS-T (0.02 M Tris-HCl, pH 7.6, 0.0137 M NaCl, and 0.1% (w/v) Tween 20) (all reagents from Sigma-Aldrich, Saint Louis, MO, USA) at RT for 1 h. Membranes were then incubated with an appropriate primary antibody (Cell Signaling Technology, Boston, MA, USA) dissolved in 5% BSA in TBS-T at 4°C overnight. Primary antibodies included a 1:1000 dilution of a rabbit anti-IL-6 antibody (catalog number 12153), a rabbit anti-GP130 antibody (catalog number 3732), a mouse anti-STAT3 antibody (catalog number 9139), a rabbit anti-Mcl-1 antibody (catalog number 94296), a rabbit anti-EGFR antibody (catalog number 4267), a phosphospecific rabbit anti-STAT3 (Tyr705) antibody (catalog number 9145), phosphospecific rabbit anti-EGFR antibodies (Tyr1045 (catalog number 2237) and Tyr1068 (catalog number 3777)), a phosphospecific rabbit anti-ERK1/2 (Thr202/Tyr204) antibody (catalog number 4370), or a phosphospecific rabbit anti-Akt (Ser473) antibody (catalog number 4060), and a 1:10,000 dilution of a mouse anti-β-actin antibody (catalog number MA1115) (Boster Biological Technology, Pleasanton, CA, USA). After three washes with TBS-T, membranes were washed and incubated with secondary antibodies (LI-COR Biosciences, Lincoln, NE, USA); an anti-mouse IgG conjugated with IRDye®800CW (catalog number 926-32210) (1:5000) or an anti-rabbit IgG conjugated with IRDye®680RT (catalog number 926-68071) (1:5000) at RT for 2 h. The immunoreactive bands were visualized using an Odyssey ® CLx Imaging System (LI-COR Biosciences, Lincoln, NE, USA). The bands were analyzed using Image Studio Lite software.

### 4.7. Immunofluorescence Study

Immunofluorescence was performed to determine NF-κB nuclear localization (upon EGF and TNF-α (PeproTech, Rocky Hill, NJ, USA) stimulation), EGFR expression and phosphorylation upon EGF stimulation, nuclear Ki-67 protein (antibody catalog number 9129, Cell Signaling Technology, Boston, MA, USA) expression, and STAT3 phosphorylation in the nucleus upon IL-6 stimulation. HeLa cells grown on glass cover slips were treated with KP extract at different concentrations (0–15 μg/mL) for 24 h in serum-free medium. Cells were stimulated with 100 ng/mL of EGF for 2 min (for pEGFR staining), for 15 min (for pERK1/2 staining), with 100 ng/mL of EGF or 20 ng/mL of TNF-α for 15 min (for NF-κB staining) (antibody catalog number 8242, Cell Signaling Technology, Boston, MA, USA), with 100 ng/mL of EGF for 24 h (for Ki-67 staining), or with 50 ng/mL of human IL-6 for 24 h (for pSTAT3 staining). After treatment, cells were fixed with 4% paraformaldehyde (Sigma-Aldrich, Saint Louis, MO, USA) (dissolved in PBS for 15 min at RT. Cells were then washed three times with PBS for 5 min, each time. Next, cells were permeabilized with 0.3% Triton X-100 in PBS for 5 min. The cells were washed three times with PBS and then blocked with 1% bovine serum BSA in PBS for 1 h. Cells were incubated with appropriate primary antibodies (Cell Signaling Technology, Boston, MA, USA) overnight at 4°C. Primary antibodies included a rabbit anti-EGFR antibody (1:50), a rabbit anti-NF-κB antibody (1:400), a rabbit anti-Ki-67 antibody (1:400), a phosphospecific rabbit anti-EGFR (Tyr1068) antibody (1:800), a phosphospecific rabbit anti-ERK1/2 (Thr202/Tyr204) antibody (1:200), and a phosphospecific rabbit anti-STAT3 (Tyr705) antibody (1:100). Cells were washed three times and incubated with appropriate secondary antibodies (a 1:500 dilution of Alexa488-conjugated goat anti-rabbit IgG, Alexa594-conjugated goat anti-rabbit IgG, or Alexa488-conjugated goat anti-mouse IgG antibodies (Thermo Fisher Scientific, USA) plus 10 µg/mL of Hoechst 33342 (Sigma-Aldrich, USA) (for nuclear staining) for 2 h, in the dark, at RT. In some experiments, Phalloidin-TRITC (1:5000) (Sigma-Aldrich, Saint Louis, MO, USA) was used to visualize filamentous actin (F-actin) (Sigma-Aldrich, Saint Louis, MO, USA). After washing three times with PBS (5 min each) and one time with distilled water (5 min), sample cover slips were mounted with Fluoromount-G (SouthernBiotech, Birmingham, AL USA). Observations were performed on a fluorescence microscope, AX70 Olympus R, Japan, with 40× magnification, and micrographs were captured with the DP-BSW Basic Software for the DP71 microscope digital camera.

### 4.8. Cell-Counting Assay 

HeLa cells were seeded in 24-well plates at a density of 2.5 × 10^4^ cells/well in complete media and incubated for 24 h at 37 °C, 5% CO_2_. Cells were then treated with KP extract at various concentrations (0, 3.75, 7.5, and 15 µg/mL), with or without the presence of EGF at 100 ng/mL. The total number of cells were counted using a haemacytometer at different time points which were at 0, 24, 48, 72, and 96 h. Three individual experiments were performed, with three replicates for each experiment.

### 4.9. Statistical Analysis

Data were analyzed by one-way analysis of variance (ANOVA). Data are presented as mean ± SD. In all analyses, a *p*-value (*p* < 0.05) was considered as statistically significant. 

## 5. Conclusions

We previously reported that KP is able to suppress growth and survival signal transduction pathways in HeLa cervical cancer cells. However, many aspects related to the anti-cancer properties of KP remain largely unexplored. Recently, accumulated evidence has elucidated the roles of the inflammatory cytokine IL-6 in the pathogenesis of HPV-positive cervical cancers. Our current study shows that KP extract can suppress the cellular expression and secretion of IL-6 protein and STAT3 activation in HeLa cells induced by EGF. The suppression of EGF-induced IL-6 production has been proven to be caused, at least in part, by the ability of KP to inhibit the phosphorylation of the EGF receptor. Consistent with the finding that KP can suppress EGFR activation, KP has been proven to possess anti-proliferative properties against HeLa cervical cancer cells. In contrast, EGF does not activate NF-κB signaling, and KP has no effect on the activation status of NF-κB, ruling out the possibility that KP decreases IL-6 production via suppressing NF-κB activation. Finally, we add additional information that KP can also inhibit autocrine IL-6 signaling, which is a key process of tumorigenesis.

## Supplementary Materials

Supplementary materials can be found at https://www.mdpi.com/1422-0067/20/17/4226/s1.

## Abbreviations

DMSO	Dimethyl sulfoxide
EGF	Epidermal growth factor
EGFR	Epidermal growth factor receptor
ELISA	Enzyme-Linked Immunosorbent Assay
GP130	Glycoprotein 130
HPLC	High Performance Liquid Chromatograph
IL-6	Interleukin 6
KP	*Kaempferia parviflora*
STAT3	Signal transducers and activators of transcription 3
TNF-α	Tumor necrosis factor-alpha
