# Supplementary material for: Kaempferia parviflora Extract Inhibits STAT3 Activation and Interleukin-6 Production in HeLa Cervical Cancer Cells
Source: Int J Mol Sci. 2019 Aug 29;20(17):4226. doi: 10.3390/ijms20174226 (PMC6747281; doi:10.3390/ijms20174226)
Supplement: Supplementary file 1 [file ijms-20-04226-s001.pdf]

# Supplementary Figures

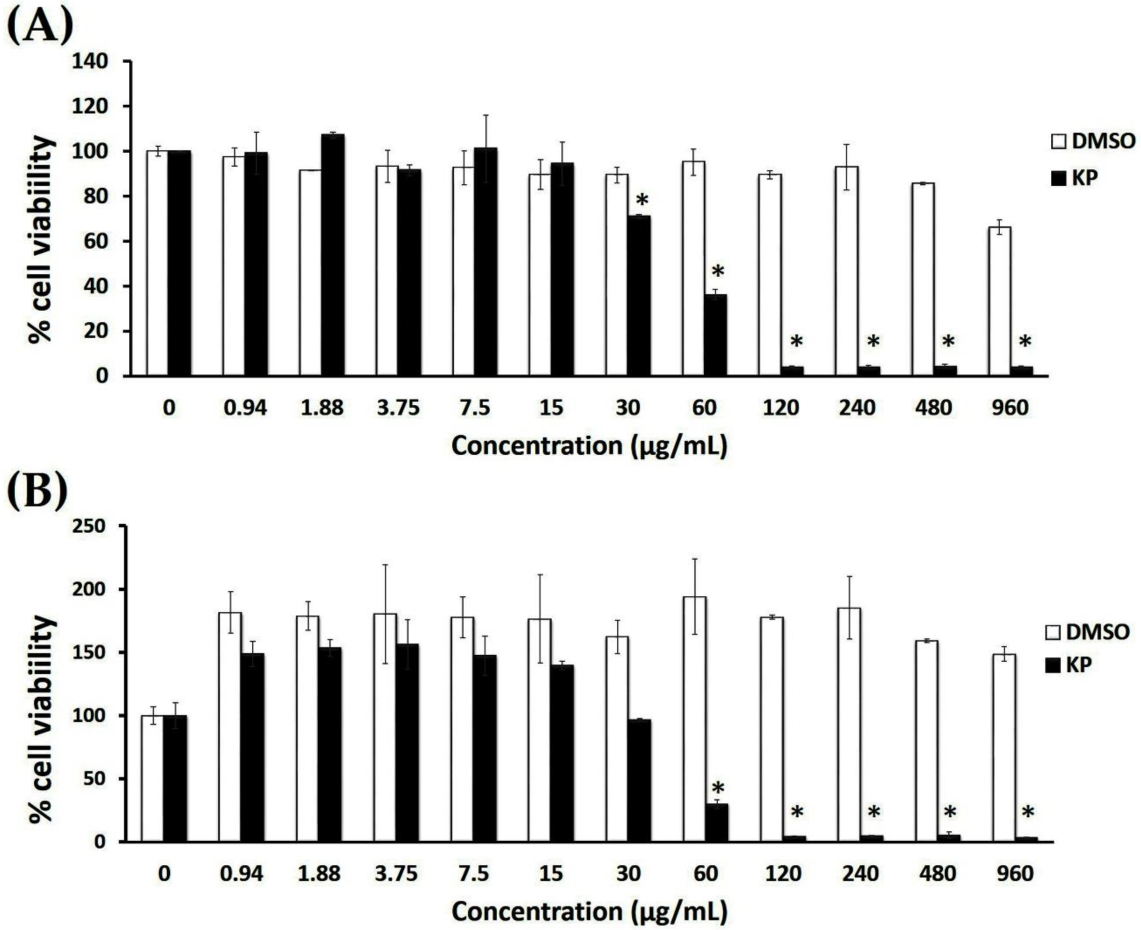


**Figure S1**. The cytotoxicity effect of different concentrations of KP ethanol extract on HeLa cells without EGF (**A**) and with 100 ng/mL EGF (**B**). DMSO was used as a vehicle control All data were from 3 independent experiments and reported as means ± SD of each quadruplicate $*p < 0.05$ compared to the untreated control (0 μg/mL).

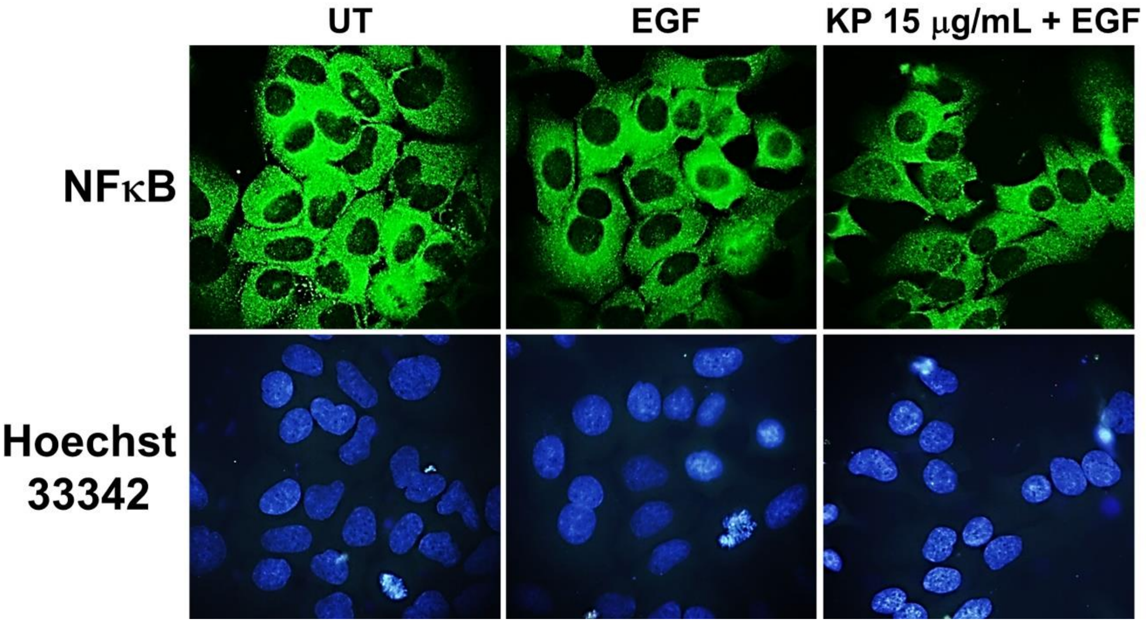


**Figure S2**. The effects of KP on nuclear localization of NF-κB in HeLa cells (**A**) Representative images from immunofluorescence study showing NF-κB staining (green) in untreated cells (UT), EGF-treated for 24 h, or HeLa cells treated with KP at 15 μg/mL and EGF for 24 h. Nuclei (blue) were counterstained with 10 μg/mL of Hoechst 33342. Micrographs were captured at 40X magnification. Data are representatives of three replicates.
